# Burden of asymptomatic malaria in adult sub-Saharan migrants attending an outpatient clinic in Rome from February 2024 to January 2025

**DOI:** 10.1186/s40249-025-01379-5

**Published:** 2025-10-23

**Authors:** Francesca Faraglia, Serena Vita, Dimitra Kontogiannis, Tommaso Ascoli Bartoli, Silvia Rosati, Nazario Bevilacqua, Gaetano Maffongelli, Angela Corpolongo, Barbara Bartolini, Antonella Vulcano, Alessandra D’Abramo, Carla Fontana, Emanuele Nicastri, Mobile-INMI group

**Affiliations:** 1 National Institute for Infectious Diseases “Lazzaro Spallanzani”, IRCCS (Istituto di Ricovero e Cura a Carattere Scientifico), Via Portuense 292, 00149 Rome, Italy; 2 Department of System Medicine, Tor Vergata University, Rome, Italy

**Keywords:** Asymptomatic malaria, *Plasmodium*, Screening, Migrant health, Infectious disease, Surveillance

## Abstract

**Background:**

Sub-Saharan African (SSA) migrants may carry asymptomatic *Plasmodium* infections after leaving endemic regions, potentially posing public health challenges in host countries. However, data on the prevalence and persistence of infection after migration are currently limited. The objective of this study is to assess the burden of asymptomatic malaria among adult SSA migrants attending an outpatient clinic in Rome.

**Methods:**

Between February 2024 and January 2025, SSA migrants attending the Lazzaro Spallanzani Institute’s mobile population clinic in Rome, Italy were screened for malaria using rapid diagnostic tests (RDTs), loop-mediated isothermal amplification (LAMP), thick smears, and real-time polymerase chain reaction (RT-PCR). Eligibility criteria included age ≥ 16, absence of fever, and origin or transit through malaria-endemic regions. Descriptive statistics were used to summarize epidemiological, clinical, and laboratory data. Continuous variables were expressed as means ± SD or median (IQR), and categorical variables as counts and percentages. A *P*-value < 0.05 was considered significant.

**Results:**

Among 87 asymptomatic migrants, malaria prevalence was 6%, all in male SSA migrants. One mixed infection was confirmed by microscopy. LAMP detected *Plasmodium* spp. in 5 cases (6.0%), confirmed by RT-PCR, while RDT identified only 2 (2.3%). Species identified by RT-PCR included *P. falciparum*, *P. malariae*, *P. ovale*, and one mixed infection. The longest time since arrival among positives was 181 days; including detention in Libya, the median interval since departure from endemic areas exceeded 600 days.

**Conclusions:**

Asymptomatic malaria can last for months after arrival and is often missed by RDTs. Incorporating RT-PCR diagnostics into routine screenings can improve early detection, lower local transmission risk, and enhance health outcomes.

**Graphical abstract:**

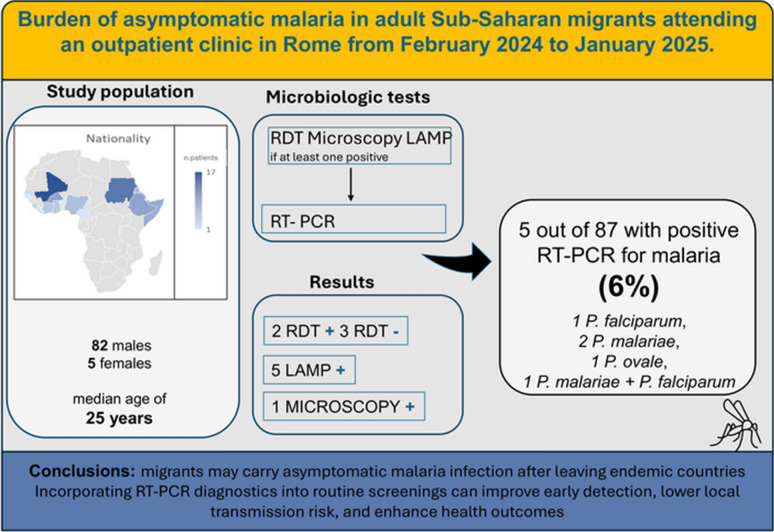

**Supplementary Information:**

The online version contains supplementary material available at 10.1186/s40249-025-01379-5.

## Background

Malaria remains a global health challenge, especially in the African Region, which accounted for 89.7% of the 263 million cases and 597,000 deaths worldwide in 2023 [1]. Repeated exposure leads to partial immunity, with many harbouring asymptomatic infections that sustain transmission and hinder elimination efforts [2]. Asymptomatic migrants from malaria-endemic regions may carry these infections in non-endemic countries, with prevalence rates ranging from 3% to 30% [3, 4]. Despite the risks, routine screening in Europe is not widely implemented, unlike in countries such as Australia and the United States [5, 6]. A Swedish study found a 9% overall prevalence rate using a screening policy based on molecular assay, detecting higher rates among Ugandan migrant adults and children [7]. This highlights the importance of carefully screening individuals arriving from areas with high rates of endemic diseases.

The study aimed to evaluate the prevalence of malaria parasites in asymptomatic migrants from sub-Saharan Africa (SSA) who attended the outpatient clinic for mobile populations at the Lazzaro Spallanzani National Institute for Infectious Diseases in Rome, Italy.

## Methods

### Study design and study population

Since February 2024, an adult outpatient clinic at the Lazzaro Spallanzani National Institute for Infectious Diseases has been dedicated to serve migrants, especially the mobile population residing in urban and suburban areas of Rome. The clinic is conducting an infectious disease screening program (February 2024–January 2025), including tests for *Schistosoma*, hepatitis B and C virus (HBV and HCV), and human immunodeficiency virus (HIV), and interferon gamma release assay (IGRA) test. Malaria screening with rapid diagnostic test (RDT), loop-mediated isothermal amplification (LAMP), thick blood smear, and real-time polymerase chain reaction (RT-PCR) assay was offered to asymptomatic patients from endemic regions in SSA or those with migration routes through these areas, regardless of their duration of residence in Italy. Participants gave informed consent, with translators’ assistance when necessary. Exclusion criteria included origin from non-endemic regions, age under 16, and fever at the time of testing.

Patients with positive results were referred to the in-patient clinic for evaluation and antimalarial drug therapy. During the clinical visit we collected data about the origin country, date of departure, migration route, duration of migration, method of travel to reach Italy, time spent in detention centres if the case, arrival date in Italy, co-morbidities and medications, previous malaria infections, specific treatments, as well as any persistent symptoms at the time of evaluation.

### Detection of malaria parasites

Malaria screening was conducted using a combination of antigen-based RDTs, microscopy, and LAMP, while RT-PCR was subsequently performed for confirmation.

RDTs (CareUs Malaria Rapydtest^®^ Combo Pf/PAN, Wells Bio, Inc., Seoul, Republic of Korea, distributed in Europe by Apacor Ltd. Wokingham, United Kingdom), were performed on sampling day to detect *P. falciparum*, *P. vivax*, *P. malariae*, or *P. ovale*. If positive for non-*P. falciparum* species, an additional rapid test (Screen Test Malaria P.F./P.V. rapid test; Screen Italia, Perugia, Italy) was conducted to differentiate *P. vivax*.

For samples that tested negative by RDT, DNA extraction was performed on the blood cell fraction and analyzed using the Alethia^®^ Malaria assay (Meridian Diagnostics, Cincinnati, OH, USA), a LAMP method for rapid detection of *Plasmodium* spp. Samples that were positive by either RDT or LAMP underwent further testing with a real-time PCR assay (Malaria Differentiation REAL TIME PCR Detection Kit—Certest Biotec^®^, Zaragoza, Spain) to identify specific *Plasmodium* species, including *P. falciparum, P. vivax, P. ovale, P. malariae,* and *P. knowlesi*.

### Data analysis

Descriptive statistics were used to summarize epidemiological, clinical, and laboratory data. Continuous variables with normal distribution were expressed as means ± standard deviation (SD), while non-normally distributed variables were reported as medians with interquartile ranges (IQR). Categorical variables were presented as counts and percentages. Statistical analyses were performed using JASP (version 0.18.3; University of Amsterdam, Amsterdam, The Netherlands).

## Results

From February 2024 to January 2025, a total of 94 participants from SSA countries attended the mobile population clinic. Five declined testing and were excluded from the study.

**Fig. 1:**
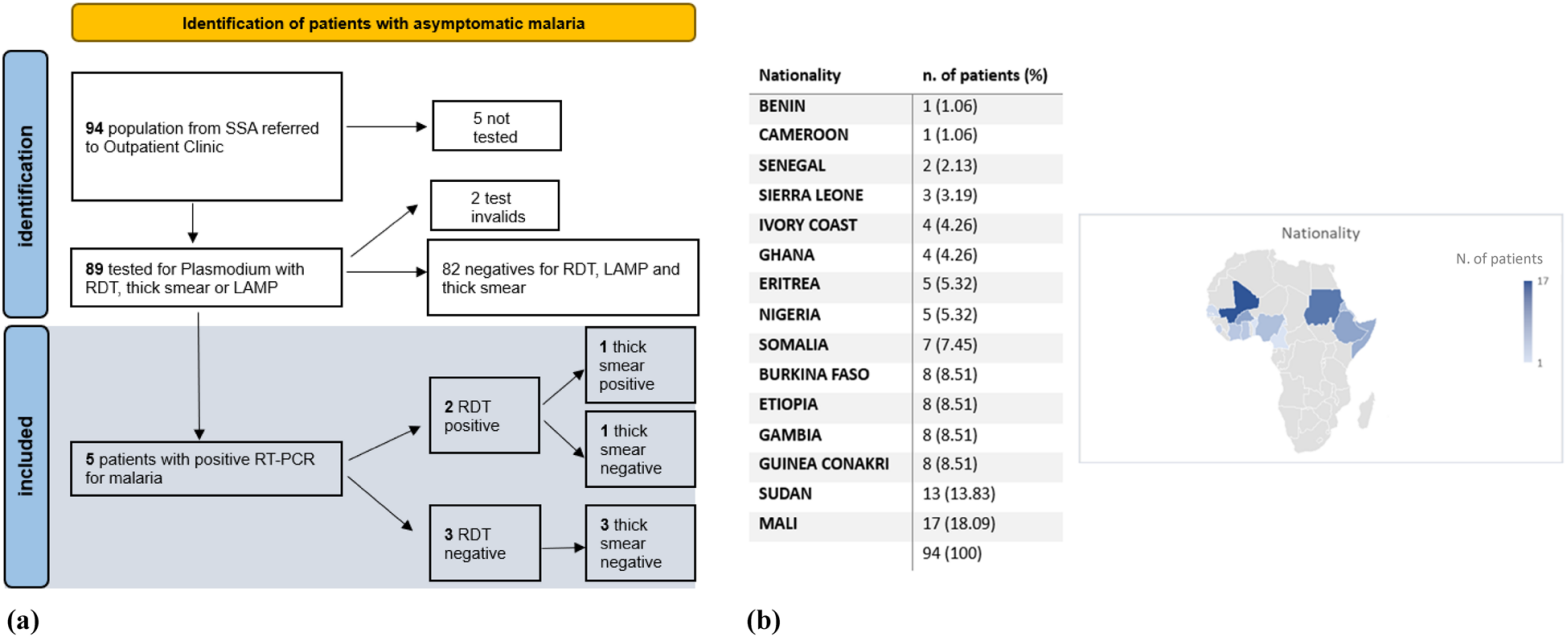
**a** Flowchart illustrating the identification process of patients with asymptomatic malaria. The diagram shows the screening and diagnostic pathway, including malaria rapid diagnostic test (RDT), microscopy, loop-mediated isothermal amplification (LAMP), and confirmatory RT-PCR testing; **b** Nationalities of the study population. The figure presents the number and percentage of participants from each country of origin in a table, accompanied by a world map highlighting their geographic distribution.

Among the 89 migrants tested, all underwent RDT, thick blood smear, and LAMP for *Plasmodium* spp (Fig. 1a), two had invalid test results and could not be followed up as they had left the city. Patients who were positive by either RDT or LAMP underwent further testing with RT-PCR.

The most represented countries were Mali (17, 18.1%) and Sudan (13, 13.8%). Males accounted for 82 participants (87.2%), with a median age of 25 years (Fig. 1b). The median time from arrival in Italy to the first screening visit was 143 days (IQR: 115–154) (Supplemental materials 1).

LAMP detected *Plasmodium* spp. in 5 of 83 patients (6.0%), while RDTs were positive in 2 of 87 cases (2.3%) (Supplemental materials 1), and both RDT-positive samples were RT-PCR confirmed. RT-PCR identified one case of *P. falciparum* (1/5, 20%), one mixed P*. falciparum*/*malariae* (1/5, 20%), one *P. ovale* (1/5, 20%), and two *P. malariae* (2/5, 40%). The mixed infection was the only case confirmed by thick blood smear (Table 1). RDT demonstrated a sensitivity of 40% (95% *CI*: 5.27–85.34) and a specificity of 100% (95%*CI*: 95.55–100) in asymptomatic patients.

**Table 1:** Demographic, clinical, and laboratory characteristics of patients with asymptomatic malaria, focusing on malaria diagnostic test results

Patient	Sex	Age, years	Nationality	Days from arrival in Italy to visit	Days from arrival in Libia to visit	RDT	Thick blood smear	RT-PCR malaria	Therapy	Schisto Ab	IGRA test	HBsAg/ HBsAb/HBcAb
1	Male	30	Benin	181	NA	Positive	Negative	*P. falciparum*	DHA/PPQ	Positive	Positive	− / − / +
2	Male	37	Sudan	7	589	Negative	Negative	*P. malariae*	HCQ	Positive	Positive	− / − / +
3	Male	29	Mali	115	660	Negative	Negative	*P. malariae*	DHA/PPQ	Positive	Negative	− / − / +
4	Male	41	Ivory Coast	143	184	Negative	Negative	*P. ovale*	DHA/PPQ primaquine	Negative	Positive	− / − / +
5	Male	22	Mali	141	1784	Positive	Positive (67 trophozoites/mmc 0.001%)	*P. falciparum* + *P. malariae*	DHA/PPQ	Negative	Negative	− / ±
Median value (IQR)	NA	30 (29–37)	NA	141 (115–143)	624 (488–941)	NA	NA	NA	NA	NA	NA	NA

*RDT* malaria rapid diagnostic test, *RT-PCR* Real-time polymerase chain reaction, *IGRA* Interferon gamma release assay, *HBsAg* Hepatitis B surface antigen, *HBcAb* Hepatitis B core antibody,* NA* not available, *DHA/PPQ* piperaquine/dihydroartemisinin, *HCQ* hydroxychloroquine

The characteristics of the five patients are illustrated in Table 1. They were all male subjects with a median age of 30 years-old (IQR: 29–37). Four of them had travelled through Libya, where they remained detained for extended periods, with a median duration of 624 days (IQR: 488–941). The median time of arrival in Italy was 141 days (IQR: 115–143), similar to the median time of the whole population (143 days, data provided in the Supplementary Material). The longest residence in Italy (181 days) was reported in the *P. falciparum* case, while the shortest (7 days) was in a *P. malariae* infection. Including the time spent in Libya, a malaria-free country since 2015, the median duration since leaving malaria-endemic regions was 624.5 days.

In patients with malaria, no laboratory abnormalities in white blood count, heamooglobin level, renal and liver function were found (data not shown). Screening for other infectious diseases revealed that three subjects were positive for IGRA with no evidence on chest X-rays and smear tests of active tuberculosis. Three individuals showed positive serology for *Schistosoma* spp. and received antiparasitic treatment. Four had evidence of prior HBV infection (HBsAg-negative, HBcAb IgG-positive, anti-HBs-negative), while one had been vaccinated against HBV. HIV and HCV serology results were negative for all participants. All five subjects were referred to the Clinical Department of the Spallanzani Institute for antimalarial treatment (Table 1).

## Discussion

In our study, a 6% prevalence rate of malaria infection was observed among asymptomatic male migrants from SSA countries, in line with other studies[7, 8]. The speices *P. malariae*, followed by *P. falciparum* and *P. ovale,* were the most prevalent*.* The longest residence in Italy among positive cases was 181 days with a median parasite survival of approximately five months, but extended to 18 months when including detention periods in Libya. For *P. ovale*, the detection of hypnozoites may explain our participant’s RT-PCR—positive result, especially since primaquine was not given. Asymptomatic co-infections with schistosomiasis and latent tuberculosis, and previous HBV exposure are reported, reflecting the endemic nature of these infections in their origin countries[9–12]. This study did not include a pediatric population aged < 16 years old, as our hospital is an adult-only facility. Consequently, the absence of data from children, who may have different exposure patterns and parasite carriage rates, could have resulted in an underestimation of the overall parasite prevalence.

The persistence of *Plasmodium* infection in humans is not well-documented, with studies reporting durations from 6 to 386 days for blood-stage infections of *P. falciparum *[7, 13]. Asymptomatic carriers, despite lacking typical febrile symptoms, act as reservoirs and can facilitate parasite transmission in non-endemic regions with suitable vectors, posing significant challenges to malaria control efforts.

In Europe, where approximately 8000 imported cases are reported annually, the risk of transmission by gametocyte carriers arriving from endemic regions remains a significant public health concern[1, 14–16]. This is particularly relevant given the recent re-emergence of *Anopheles sacharovi* in Apulia, thought to be extinct in Italy for over 50 years, which highlights the growing receptivity of Italian southern regions [13]. Our findings suggest that these undiagnosed and unreported cases may significantly add to the total number of infected individuals, representing a hidden reservoir for potential transmission. Despite the small sample size and limited number of positive cases, the prevalence of asymptomatic *Plasmodium* infections observed in our study is consistent with previous reports in migrant populations [7, 8]. This finding should be interpreted as exploratory and context-specific, reflecting the diagnostic performance and feasibility of molecular screening in a real-world outpatient setting. Screening strategies for malaria vary considerably across countries. In Italy, there is no formal recommendation to screen African migrants, or other population groups, for asymptomatic malaria infection. National guidelines introduced in July 2015 recommend the use of RDTs for migrants presenting with symptoms suggestive of malaria, while RT-PCR testing is reserved for high-risk groups, including pregnant women and immunocompromised individuals[17]. In our study, malaria testing by RDT and RT-PCR was performed in asymptomatic individuals exclusively within the research protocol, and not as part of routine clinical practice. Compared to microscopy and RDTs, RT-PCR testing demonstrates superior sensitivity, particularly for detecting low-level parasitaemia in asymptomatic individuals, highlighting its utility as a screening modality for migrants originating from malaria-endemic areas [3, 7]. In our cohort, the sensitivity of RDTs was 40%, consistent with other studies in asymptomatic migrants, where sensitivity ranged from 30% to 50% depending on parasite density and species [3, 4]. These findings emphasize the limited performance of antigen-based tests in low-parasitemia settings and support the integration of molecular diagnostics. However, PCR performance may be affected by low parasite densities, with possible false positives or negatives. Recent work suggests that ultrasensitive methods, such as high-volume qPCR or nested PCR, could further improve detection in this context [18, 19]. Although our study was not designed to assess transmissibility or clinical outcomes, it documents persistent asymptomatic malaria, with cases detectable more than 6 months after arrival and over 600 days since departure from endemic regions. This persistence highlights the limitations of screening windows based solely on recent travel history and supports the need for sustained and inclusive strategies.

In Italy where over 225,000 refugees and migrants arrived by sea between 2023 and 2024[20]—primarily from West and Central Africa and with more than 5 million, foreign residents and an estimated 500,000 undocumented migrants. [21–23] Such findings underscore the importance of screening programs tailored to migration routes and epidemiological risk. The active involvement of frontline healthcare workers and general practitioners is essential to effectively address the healthcare access barriers faced by asylum seekers, undocumented migrants, and, more broadly, mobile populations.

## Conclusions

This study suggests that asymptomatic *Plasmodium* infections may persist for several months after arrival in a non-endemic country, underscoring the long-term resilience of the parasite in the absence of clinical symptoms. In our sample, RDTs missed some infections that were identified by molecular methods such as RT-PCR. Although numbers were small, these findings, consistent with previous literature data, raise concerns about the sensitivity of RDTs in low-transmission settings and suggest that reliance on standard antigenic diagnostic approaches alone may lead to underdetection of chronic carriers.

Detectable infections can persist for more than six months after arrival—and over 600 days post-departure from endemic regions—indicating that standard screening periods based on recent travel may miss asymptomatic cases. Incorporating RT-PCR diagnostics into routine screenings for migrants from malaria-endemic countries can improve early detection, lower local transmission risk, and enhance health outcomes.

These findings highlight the need for ongoing, inclusive infectious disease screening policies that address mobile populations’ risk profile and migration trajectories beyond initial entry. Strengthening migration screening policies supports effective case management and public health planning in increasingly multicultural and mobile societies.

## Supplementary Information

Supplementary Material 1.Characteristics of Sub-Saharan (SSA) patients and patients with asymptomatic malaria.

## Data Availability

All data generated or analyzed relating to this study are presented within this published article.

## Abbreviations

SSA: Sub-Saharan Africa
HBV: Hepatitis B virus
HCV: Hepatitis C virus
HIV: Human immunodeficiency virus
IGRA: Interferon gamma release assay
RDT: Rapid diagnostic test
LAMP: Loop-mediated isothermal amplification
RT-PCR: Real-time polymerase chain reaction
SD: Standard deviation
IQR: Interquartile range
HBsAg: Hepatitis B surface antigen
HBcAb: Hepatitis B core antibody
anti-HBs: Hepatitis B surface antibody
