# Supplementary material for: Burden of asymptomatic malaria in adult sub-Saharan migrants attending an outpatient clinic in Rome from February 2024 to January 2025
Source: Infect Dis Poverty. 2025 Oct 23;14:107. doi: 10.1186/s40249-025-01379-5 (PMC12548191; doi:10.1186/s40249-025-01379-5)
Supplement: Supplementary file 1 — Supplementary Material 1.Characteristics of Sub-Saharan (SSA) patients and patients with asymptomatic malaria. [file 40249_2025_1379_MOESM1_ESM.docx]

|  | **Sex M/F (%)** | **Age median [IQR]** | **Days from arrival median [IQR]** | **Any test for malaria* (%)** | **RDT positive (%)** | **PCR malaria (%)** |
| --- | --- | --- | --- | --- | --- | --- |
| **Overall SSA patients** | 82/12; (87.23/12.77) | 25 [22-31] | 143 [24-395] | 87/94  (92.55) | 2/87 (2.30) | 5/83 (6.02) |
| **Patients with asymptomatic malaria** | 5/0;  (100/0) | 30 [29-37] | 141 [115-143] | 5/5 (100) | 2/5 (40) | 5/5 (100) |

**Supplemental materials 1.** Characteristics of Sub-Saharan (SSA) patients and patients with asymptomatic malaria.

*smear test and/or PCR

RDT: malaria rapid diagnostic test; PCR: Polymerase chain reaction;
